# Combining Public Opinion Dissemination with Polarization Process Considering Individual Heterogeneity

**DOI:** 10.3390/healthcare9020176

**Published:** 2021-02-07

**Authors:** Tinggui Chen, Jingtao Rong, Jianjun Yang, Guodong Cong, Gongfa Li

**Affiliations:** 1 School of Statistics and Mathematics, Zhejiang Gongshang University, Hangzhou 310018, China; rjt323@126.com; 2 Department of Computer Science and Information Systems, University of North Georgia, Oakwood, GA 30566, USA; Jianjun.Yang@ung.edu; 3 School of Tourism and Urban-Rural Planning, Zhejiang Gongshang University, Hangzhou 310018, China; cgd@mail.zjgsu.edu.cn; 4 Hubei Key Laboratory of Mechanical Transmission and Manufacturing Engineering, Wuhan University of Science and Technology, Wuhan 430081, China; ligongfa@wust.edu.cn

**Keywords:** public opinion dissemination, public opinion polarization, SEIR model, J-A model, individual heterogeneity

## Abstract

The wide dissemination of false information and the frequent occurrence of extreme speeches on online social platforms have become increasingly prominent, which impact on the harmony and stability of society. In order to solve the problems in the dissemination and polarization of public opinion over online social platforms, it is necessary to conduct in-depth research on the formation mechanism of the dissemination and polarization of public opinion. This article appends individual communicating willingness and forgetting effects to the Susceptible-Exposed-Infected-Recovered (SEIR) model to describe individual state transitions; secondly, it introduces three heterogeneous factors describing the characteristics of individual differences in the Jager-Amblard (J-A) model, namely: Individual conformity, individual conservative degree, and inter-individual relationship strength in order to reflect the different roles of individual heterogeneity in the opinions interaction; thirdly, it integrates the improved SEIR model and J-A model to construct the SEIR-JA model to study the formation mechanism of public opinion dissemination and polarization. Transmission parameters and polarization parameters are simulated and analyzed. Finally, a public opinion event from the pricing of China’s self-developed COVID-19 vaccine are used, and related Weibo comment data about this event are also collected so as to verify the rationality and effectiveness of the proposed model.

## 1. Introduction

With the rapid development of mobile Internet technology, online social platforms have attracted many users due to their openness and convenience, and users express their opinions on social hot events with the platforms. These opinions have further evolved into online public opinion through the interaction and convergence of online platforms. However, online social platforms have brought about two phenomena that cannot be ignored in the release and dissemination of information: First, the wide dissemination of false information on online platforms may strongly mislead the public’s behavior, leading to serious mass incidents and huge social influence (for example, after the outbreak of COVID-19, there were rumors that Shuanghuanglian Oral Liquid could prevent COVID-19, thus resulting in Shuanghuanglian Oral Liquid being out-of-stock in pharmacies); second, the intense collision of views among different individuals can easily lead to extreme phenomena such as online confrontation and online condemnation (such as the “Internet condemnation” triggered by the “Freud Incident”), which impact on the harmony and stability of society. The aforementioned two phenomena belong to the issue of public opinion dissemination and public opinion polarization, respectively, and the process of public opinion dissemination and polarization often interoperates in terms of influence and promotion, making their influence further expanded. If the public opinion polarization and dissemination are not combined, and only one of the two is selected for research, it will not be able to fully explain the internal mechanism of the evolution of public opinion. Based on this, it has important theoretical and practical significance to combine the issues of public opinion polarization and public opinion dissemination for in-depth research.

At present, scholars have conducted a lot of research and made achievements on the polarization and dissemination of public opinion. Generally speaking, the research on the former can be divided into two categories: (1) Qualitative analysis of the phenomenon of public opinion polarization from the perspective of the phenomenon itself. These studies mainly study the causes of polarization of public opinion and corresponding counter measures. However, most of qualitative studies are short of specific empirical investigations and quantitative methods, and thus turn out subjective conclusions. In addition, they don’t explain the complex evolution of public opinion; (2) quantitative analysis of the phenomenon of public opinion polarization from the perspective of system dynamics. Common models include the Sznajd model [1], Voter model [2], Deffuant-Weisbuch model (D-Wmodel) [3], and Jager-Amblard model (J-A model) [4]. Although the above quantitative models can reveal the local characteristics of polarization, they cannot accurately reflect the complex and changeable polarization of public opinion. In addition, most of the research on the dissemination of public opinion is based on the analysis of infectious disease models. Commonly used models are: Susceptible-Infected model (SI model) [5], Susceptible-Infected-Susceptible model (SIS model) [6], Susceptible-Infected-Recovered model (SIR model) [7], and Susceptible-Exposed-Infected-Recovered model (SEIR model) [8]. In these models, the nodes in the network are usually regarded as ordinary individuals in reality and are further subdivided into several categories. Different categories of individuals represent different states they hold during the dissemination process. For example, in the SEIR model, network nodes are subdivided into uninformed individuals, silent individuals, communication individuals, and immune individuals according to their states. In fact, the dissemination and polarization processes of public opinion are often carried out simultaneously and influence each other. Most likely, existing studies choose one of the two for analysis, but rarely combine the two for comprehensive analysis. Although Chen et al. [9] combined the dissemination of public opinion with the dissemination process, the model in the article did not further consider the connection between the dissemination and the process of polarization. Based on this, this article combines the infectious disease model with the opinion interaction model and introduces individual heterogeneity factors such as individual communicating willingness, forgetting effect, individual conformity, individual conservative degree, and inter-individual relationship strength to construct the SEIR-JA model combining the polarization and dissemination process of public opinion. Finally, this article stimulates public opinion evolution process through simulation experiments and analyzes the influence of public opinion evolution from the perspective of model parameters and network structure.

The structure of the paper is organized as follows: Section 2 is literature review; Section 3 builds a SEIR-JA model that integrates the dissemination and polarization of public opinion; Section 4 simulates the evolution of public opinion through simulation experiments, and studies the impact of model parameters on the dissemination and polarization of public opinion; Section 5 verifies the SEIR-JA model with actual cases; Section 6 is a summary of the full text and prospects for future work.

## 2. Literature Review

This section reviews related literature from two aspects: Public opinion dissemination and public opinion polarization.

For the research of public opinion dissemination, most of the existing literature uses infectious disease models to analyze the process of public opinion dissemination. For example, Kyrychko and Blyuss [10] derived and studied a delayed SIR model with a general incidence rate. The time delay represented the temporary immunity period, that was, the time from recovery to sensitivity. In this study, both trivial and endemic equilibria were found, and their stability was investigated. Zhang and Zhu [11] studied two kinds of rumor dissemination dynamics with quadratic relationship by establishing the I2S2R model, and concluded that the dissemination intensity of second rumors depended on the dissemination intensity of initial rumors. Based on the SIR model, Jiang and Yan [12] proposed a piecewise SIR model to quantify the dissemination speed, scale, and influence of online information. The simulation results showed that there was no a proportional relationship between the sustained influence of a message and the number of disseminators. Kabir et al. [13] suggested that individuals in a population could be classified into six states as unaware susceptible (SU), aware susceptible (SA), unaware infected (IU), aware infected (IA), unaware recovered (RU), and aware recovered (RA). They incorporated all possible states of unaware–aware (UA) with SIR process and established the SIR-UA model. Zan et al. [14] considered the counter attack mechanism of rumor dissemination and introduced the SICR model and the adjusted SICR model to study the influence of self-resistance parameter *τ* on rumor propagation. The SICR model was compared with SIR model and adjusted SICR model, and the dissemination peak and final size of rumors with various parameters were analyzed. Wu and Gergely [8] proposed SEIR model, in which the infection time depended on the distribution of infection age and had infinite delay. Zhu et al. [15] introduced user similarity, information value, and information timeliness to build an improved SEIR model. Simulation experiments showed that the model could better explain the influence of relevant influencing factors on WeChat information dissemination. Dong et al. [16] established an SEIR rumor dissemination model to describe the online social network with a varying total number of users and user deactivation rate. The simulation results indicated that the SEIR model of rumor dissemination in online social network with changing total number of users could accurately reveal the inherent characteristics of rumor dissemination process in online social network. Most of the aforementioned literatures have added more diverse individual states on the basis of classic infectious disease models. However, since the psychological factors of individual state transition are not considered, most studies still describe individual state transition with fixed probability. In fact, in the process of individuals participating in the discussion of hot events, individual psychological factors often determine the individual’s state, and the large-scale dissemination of public opinion is usually the result of further evolution based on the transformation of individual state. Based on this, in order to reveal the formation mechanism of public opinion dissemination, it is necessary to conduct in-depth research on the psychological factors of individual state transition.

For the research on the phenomenon of public opinion polarization, most of the existing literature uses the opinion interaction model to analyze the polarization process. Generally, opinion interaction models can be divided into discrete models and continuous models. Discrete models mainly include Voter model and Sznajd model, etc., which are suitable for the decision-making of simple binary discrete opinions such as individual agreement or disapproval, which cannot specifically describe the process of opinion change. The continuous model is based on the bounded confidence assumption, the representative ones are the D-W model and the J-A model. In the D-W model, when the agents’ attitude difference is lower than the given threshold, the agents will adjust the attitude according to the interaction. The J-A model is based on the D-W model, adding neutral and repulsive behavior to the process of opinion interaction, so this model is more in line with the interaction mechanism of opinions between individuals in the real world. At present, many scholars have used continuous models to conduct a lot of research on the phenomenon of public opinion polarization. For example, Zhang and Hong [17] proposed and analyzed two generalized Deffuant-Weisbuch (D-W) models named SMDW model and LMDW model. Mare and Latora [18] considered that the individuals had different inclinations to change opinion and different abilities in convincing the others andobtained the so-called “Stubborn individuals and Orators” (SO) model. Lorenz [19] changed the uniform bounds of confidence in the traditional D-W model to the heterogeneous bounds of confidence. Simulation results showed that a society of agents with two different bounds of confidence (open-minded and closed-minded agents) could find consensus even when both bounds of confidence were significantly below the critical bound of confidence of a homogeneous society. Carro et al. [20] studied the influence of initial distribution of agent attitude on the final state of opinion evolution in D-W model. Simulation results showed that under the bounded confidence interaction rules, agents could be promoted or prevented from reaching consensus by changing the initial distribution of attitude.Based on the social judgment theory, Chau et al. [21] extended the J-A model and established a general model of opinion formation with isomorphic subjects. By combining the classical J-Amodel, Chen et al. [22] proposed a multidimensional opinion evolution model for studying the dynamics of opinion polarization. Liang et al. [23] proposed a discrete-time model of opinion dynamics. They investigated the influence of heterogeneity in confidence distribution and influence distribution on the interactive behavior, which has shown that heterogeneity did not always promote consensus, and there was an optimal heterogeneity so that the relative size of the largest consensus cluster reached the maximum in heterogeneous confidence and influence networks. Li and Zhang [24] proposed and analyzed the heterogeneity bounded confidence model. There were three special agents in the model, infector, extremist, and leader. The infector was specified as an agent with large eyeshot, and the extremist was the agent with high confidence. The leader possessed both high confidence and large eyeshot. Results showed the system was more realistic than the classic model. Most of the above literatures divided individuals into several categories according to a single heterogeneity factor. However, the differences among individuals are diverse and complex. So, a single heterogeneity factor cannot well reflect the role of heterogeneity factors in the process of public opinion polarization. Based on this, a variety of heterogeneous factors should be considered in the research of public opinion polarization.

To sum up, the current academic group has done in-depth research on the dissemination and polarization of public opinion. However, a comprehensive analysis of the two is rarely done, and usually only focuses on one type of problem. In reality, the process of public opinion dissemination and polarization often proceed simultaneously and influence each other. Therefore, in view of the shortcomings of existing research, this paper integrates the process of public opinion dissemination and polarization and builds the SEIR-JA model based on the improved dissemination model and opinion interaction model. This model comprehensively considers the process of public opinion disseminationand polarization, shows the evolution process of public opinion information, and can more accurately describe the dynamic interaction process of netizens’ opinions, so it has good applicability.

## 3. Model Construction

**Figure 1:**
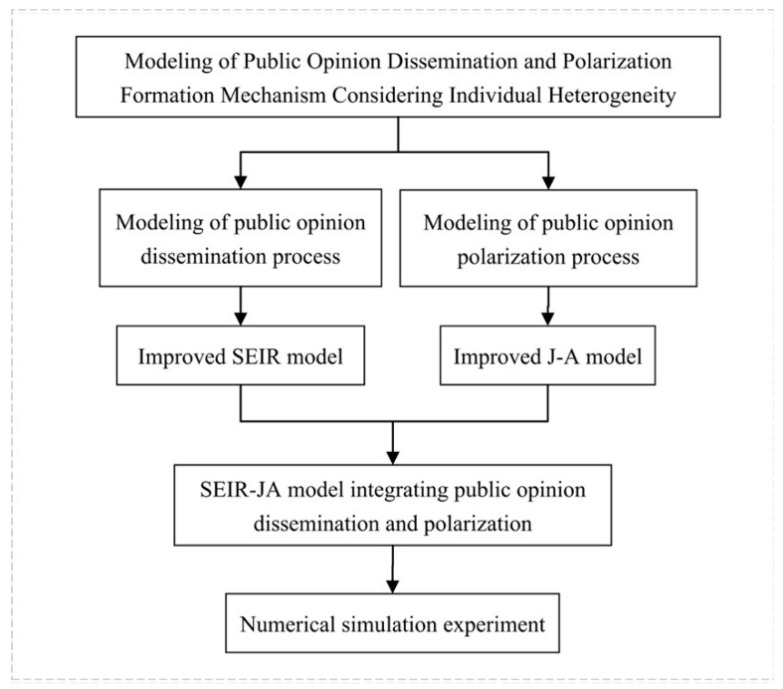
Research idea.

Although the SEIR model and the J-A model are widely used, they ignore the role of individual heterogeneous characteristics in the dissemination and polarization of public opinion. In addition, both the SEIR model and the J-A model only focus on one of the processes, thus it is difficult to explain the connection between the dissemination and polarization of public opinion. Based on this, this section improves the deficiencies of the SEIR model and the J-A model firstly. Then it integrates the improved SEIR model and the J-A model to construct the SEIR-JA model. The research idea of the paper is shown in Figure 1:

The parameters and variables involved in the model are shown in Table 1 and Table 2:

### 3.1. Modeling the Process of Public Opinion Dissemination

The SEIR model uses a fixed probability to describe individual state transitions, simply homogenizes all individuals, and ignores the heterogeneous characteristics of individuals, which cannot explain the internal mechanism of individual state transitions in detail. Aiming at the deficiencies of the SEIR model, an improved SEIR model is constructed. Like the traditional SEIR model, the improved SEIR model divides the people involved in the discussion of hot events into four categories: Uninformed individuals, silent individuals, communication individuals, and immune individuals. Among them, uninformed individuals represent individuals who have not received public opinion information; silent individuals represent individuals who have received public opinion information, but have not diffused it to uninformed individuals; communication individuals represent individuals who have received public opinion information and diffused it to uninformed individuals; and immune individuals refer to individuals who are no longer interested in public opinion information in the dissemination of public opinion.

The improved SEIR model introduces two individual heterogeneity factors, namely: The individual communicating willingness and the forgetting effect, and uses them as a condition for individual state transition.

(1) The individual communicating willingness. It refers to the tendency to initiate dissemination when given the opportunity [25], evaluating whether individuals can externalize to express, which is the important factor for diffusing public opinion in social network. Generally speaking, the factors that affect the individual communicating willingness can be summarized in two aspects: One is the extreme degree of individual opinion, that is, the more extreme an individual’s opinion is, the stronger the communicating willingness in the network will be; the other is the external recognition degree of individual opinion, that is, the higher the external recognition degree of an individualopinion is, the stronger the individual communicating willingness will be. Therefore, *P_i_*(*t*) is described by the following formula [26]:

(1) $$P_{i}(t)=(\left|x_{i}(t)\right|-1)e^{1-m_{it}}+1$$

where |*x_i_*(*t*)| reflects the extreme degree of individual opinion. Adding 1 at the end and subtracting from |*x_i_*(*t*)| promise that *P_i_*(*t*) belongs to [0, 1].

(2) The forgetting effect. It refers to the phenomenon that an individual’s attention to a hot event will decay over time. The forgetting effect is described by *z* (the time length of receiving public opinion information). It is assumed that when the individual receives the public opinion information for the first time, *z* = 1, and at each subsequent moment, *z* increases by 1. At the same time, set *z*_0_ as the maximum length of time an individual pays attention to a piece of public opinion information. When an individual receives a piece of public opinion information for a time length *z* greater than *z*_0_, it is considered that he/she no longer pays attention to the public opinion information and no longer participates in opinion interaction.

**Figure 2:**
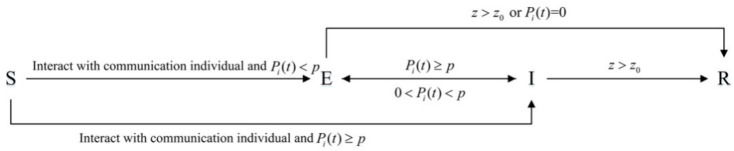
Individual state transition rules in the improved SEIR model.

In addition, the improved SEIR model also optimizes the transformation mechanism of the individual state, which is specifically embodied in the following three situations: (1) An uninformed individual is directly transformed into a communication individual; (2) a silent individual is directly transformed into an immune individual; (3) a communication individual transforms into a silent individual. At the same time, the improved SEIR model has the following settings: When an uninformed individual interacts with a communication individual, the uninformed individual will transform into a silent or communication individual according to his/her own communicating willingness; when the silent individual’s communicating willingness is greater than or equal to the communication threshold *p* (the critical value for individuals to express their opinions), he/she turns into a communication individual; if the communicating willingness of the communication individual is less than the communication threshold *p*, he/she turns into a silent individual; when an individual’s communicating willingness is less than 0 or the time of receiving public opinion information is too long, he/she turns into an immune individual. The individual state transition rules in the improved SEIR model are shown in Figure 2:

In Figure 2, S represents an uninformed individual; E represents a silent individual; I represents a communication individual; R represents an immune individual.

### 3.2. Modeling the Process of Public Opinion Polarization

The J-A model assumes that any individual has the same acceptance of the opinions of other individuals, and it does not consider the role of individual heterogeneous characteristics in the process of public opinion polarization. In fact, there are certain differences in the acceptance of other individuals’ opinions by different individuals, and this difference will have an impact on the opinions interaction. In view of the shortcomings of the J-A model, an improved J-A model is constructed. The improved J-A model introduces three individual heterogeneity factors: Inter-individual relationship strength, individual conservative degree, and individual conformity.

(1) The inter-individual relationship strength. In reality, individuals tend to trust and listen to the opinions of friends, and the opinions of close friends are more convincing than ordinary friends. Therefore, the inter-individual relationship strength will have an impact on the process of public opinion polarization, that is, the closer the relationship between individuals is, the higher the acceptance of each other’s opinions will be. Here, the concept of individual embedding degree [27], that is, the number of friends that two individuals have in the network, is used to describe the strength of the relationship between individuals, represented by Formula (2):

(2) $$E_{ij}=\{\begin{array}{cc} {\frac{n_{ij}}{\left(k_{i}-1)+(k_{j}-1\right)}}_{} & k_{i},k_{j}\neq 1\\ 1_{} & k_{i}=k_{j}=1 \end{array}$$

where *k_i_* − 1 represents the number of neighbors remaining for individual *i* except for individual *j*(*k_i_* − 1) + (*k_j_* − 1) represents the maximum number of common neighbors that may exist between individual *i* and *j*. In addition, the premise of setting the interaction between the two individuals is that the two individuals have a direct connection in the network. Therefore, when *k_i_ = k_j_* = 1, it means that individuals *i* and *j* are each other’s only neighbors, and the relationship between the two is the strongest, that is, *E_ij_* = 1.

(2) The individual conservative degree. In actual communication, people with more conservative thinking tend to be less likely to accept others’ opinions. Therefore, in the process of public opinion polarization, the higher the individual conservative degree is, the lower the acceptance of others’ opinions will be. Here it is assumed that the individual conservative degree is determined by the number of neighbors. The more individual neighbors are, the more potential public opinion information they will have, and the lower the conservative degree when interacting with other individuals will be, that is, the number of individual neighbors is negatively correlated with the individual conservative degree. The individual conservative degree is described by the following formula [28]:

(3) $$T_{i}=N\times T_{0}\times \frac{k_{i}^{-1}}{\sum_{l=1}^{N}k_{l}^{-1}}$$

**Figure 3:**
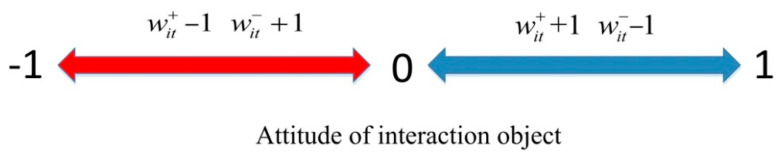
Change rules of *w_it_*^+^ and *w_it_*^−^.

(3) The individual conformity. Individual conformity refers to the phenomenon that when individuals are affected by the group, their opinions will change in the same direction as the majority [29]. Individual conformity is gradually formed in the process of public opinion polarization, which describes the dynamic change process of the individual’s acceptance of others’opinions. In order to clarify the formation process of individual conformity, the concepts of positive and negative opinions are introduced here, and it is assumed that positive opinions represent opinions with attitude values greater than 0; negative opinions represent opinions with attitude values less than 0. Since individuals cannot fully know everyone’s opinions on public opinion events, individuals’ judgments of mainstream opinions (here mainstream opinions refer to the majority of people in the network opinions) will change during the interaction. When an individual has more exposure to positive opinions (negative opinions) than negative opinions (positive opinions), the individual will think that positive opinions (negative opinions) are mainstream opinions, so that the conformity of positive opinions (negative opinions) will increase. Based on this, *w_it_*^+^ and *w_it_*^−^ are defined to, respectively, represent the mainstream degree of the positive and negative opinions which individual *i* consider at time *t* (the mainstream degree refers to the proportion of people holding a certain opinion in the network). In addition, *w_it_*^+^ + *w_it_*^−^ = 1 and *w_i_*_0_^+^ = *w_i_*_0_^−^ = 0.5 are set here. When *w_it_*^+^ > *w_it_*^−^, it means that individual *i* thinks that the positive opinion is the mainstream opinion; when *w_it_*^+^ < *w_it_*^−^, it means that the individual *i* thinks that the negative opinion is the mainstream opinion. The change rules of *w_it_*^+^ and *w_it_*^−^ are shown in Figure 3:

In Figure 3, *γ* is the change range of *w_it_*^+^ and *w_it_*^−^ per unit time, which describes the judgment of individual *i* on the change range of the mainstream opinion in a single interaction. When the interaction object holds a positive opinion (negative opinion), *w_it_*^+^ increases by *γ* units (*w_it_*^−^ decreases by *γ* units) and *w_it_*^−^ decreases by *γ* units (*w_it_*^+^ increases by *γ* units). The changes of *w_it_*^+^ and *w_it_*^−^ will further affect the conformity of individuals to positive and negative views. It is assumed here that *C_it_*^+^ represents the conformity of individual *i* to the positive opinion at time *t*, and *C_it_*^−^ represents the conformity of individual *i* to the negative opinion at time *t*, and both are expressed by Formula (4):

(4) $$\{\begin{array}{c} C_{it}^{+}=2^{w_{it}^{+}}-1\\ C_{it}^{-}=2^{w_{it}^{-}}-1 \end{array}$$

It is assumed that in the improved J-A model, *μ_it_* is determined by three heterogeneous factors: Inter-individual relationship strength, individual conservative degree, and individual conformity. *μ_it_* is expressed as follows:

(1) When *x_j_*(*t*) ≥ 0:

(5) $$\mu_{it}=(1+E_{ij})\times (1-T_{i})\times C_{it}^{+}$$

(2) When *x_j_*(*t*) < 0:

(6) $$\mu_{it}=(1+E_{ij})\times (1-T_{i})\times C_{it}^{-}$$

where 1 + *E_ij_* is to prevent *μ_it_* from approaching 0 due to being too small *E_ij_*; (1 − *T_i_*) reflects the negative correlation between the coefficient of change of opinion and individual conservative degree.

The opinion interaction rules in the improved J-A model areas follows:

(1) When |*x_i_*(*t*) − *x_j_*(*t*)| < *d*_1_:

(7) $$\{\begin{array}{c} x_{i}(t+1)=x_{i}(t)+\mu_{it}\times (x_{j}(t)-x_{i}(t))\\ x_{j}(t+1)=x_{j}(t)+\mu_{jt}\times (x_{i}(t)-x_{j}(t)) \end{array}$$

(2) When |*x_i_*(*t*) − *x_j_*(*t*)| > *d*_2_:

(8) $$\{\begin{array}{c} x_{i}(t+1)=x_{i}(t)-\mu_{it}\times (x_{j}(t)-x_{i}(t))\\ x_{j}(t+1)=x_{j}(t)-\mu_{jt}\times (x_{i}(t)-x_{j}(t)) \end{array}$$

(3) Other siuations:

(9) $$\{\begin{array}{c} x_{i}(t+1)=x_{i}(t)\\ x_{j}(t+1)=x_{j}(t) \end{array}$$

### 3.3. SEIR-JA Model Framework and Simulation Implementation

**Figure 4:**
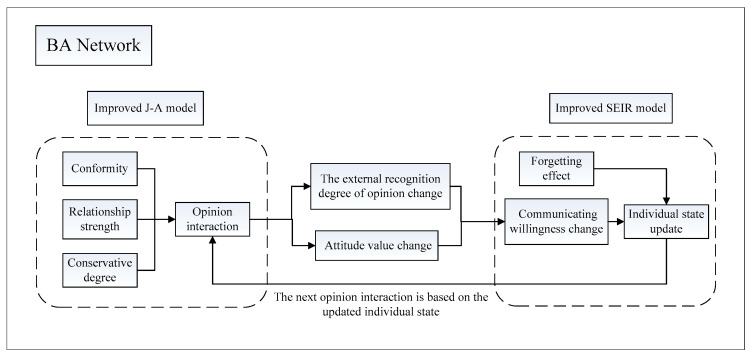
SEIR-JA (Susceptible-Exposed-Infected-Recovered–Jager-Amblard) model framework.

Based on Barabási-Albert network (BA network) [30], considering the aforementioned improved SEIR model and J-A model comprehensively and adopting Monte Carlo’s multi-agent method, a SEIR-JA model that integrates the process of public opinion dissemination and polarization is constructed to reflect the whole process of public opinion evolution. The model frame is shown in Figure 4.

It can be seen from Figure 4 that the dissemination and the polarization process of public opinion described by the SEIR-JA model are not independent of each other, but affect each other. The change of individual state at time *t* directly determines the number of communication individuals in the process of opinion interaction at time *t* + 1, and then affects the process of public opinion polarization. At any time, the change of individual attitude values and ofthe external recognition degree of opinion caused by the interaction of opinion at any time will affect the communicating willingness and thus affect the process of individual state update.

The specific simulation steps of the model are as follows:

(1) At the initial moment, a certain number of individuals are randomly selected as communication individuals. According to Equation (1), the individual communicating willingness is generated. The time length of receiving public opinion information is set as 1.
(2) At each unit moment, communication individual *i* randomly selects neighbor individual *j* as the interaction object and interacts according to the state of individual *j*. According to the different states of individual *j*, the interaction can be divided into the following two situations: (1) If individual *j* is an uninformed individual, the initial attitude value and initial communicating willingness *P_j_*(1) will be formed by individual *j* first, and then it will be transformed into a communication individual or silent individual according to the communicating willingness. Then, communication individual *i* and individual *j* interact according to Equations (7)–(9). (2) When individual *j* is a silent individual or a communication individual, communication individual *i* and individual *j* directly interact according to Equations (7)–(9).
(3) At each unit moment, after the interaction of all communication individuals, the communicating willingness, the time length of receiving public opinion information, and the state of the individuals in the network are updated.
(4) Determine whether the opinion interaction meets the end condition. The condition for ending the interaction are set as follows:

(10) $$\sqrt{\sum_{i=1}^{N}{\left(x_{i}(t)-x_{i}(t-1)\right)}^{2}}\leq 0.1$$

**Figure 5:**
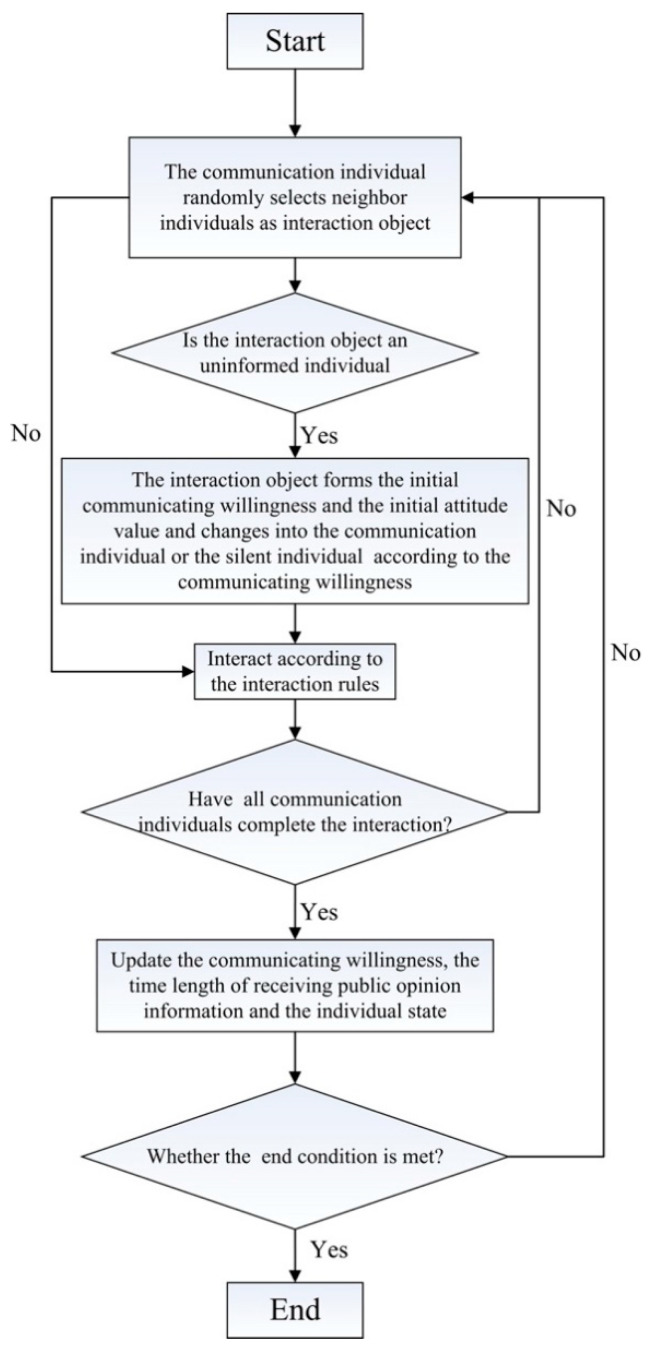
The evolution of public opinion.

If the end condition of opinion interaction is not met, steps (2)–(5) are repeated until the end condition of opinion interaction is met, and the interaction process is ended. The specific flow chart is shown in Figure 5.

## 4. Numerical Simulation Experiment

In this section, Monte Carlo Multi-Agent method is adopted to conduct comparative simulation experiments from dissemination parameters and polarization parameters to explore the influence of different factors on the evolution process of public opinion.

The initial attitude value of the individuals *x_i_*(0) obeys *N*~(0, 0.3876), and maps in [−1, 1] interval, the attitude value less than −1 is set to −1, the attitude value greater than 1 is set to 1, so that most individuals hold neutral opinion, and only a few individuals hold extreme opinion, which conforms to the reality. At the same time, BA network is selected to construct the simulation network, and the individual size in the network is set to 300.

In order not to lose generality, the SEIR-JA model is run ten times here, and then the ten results are averaged to get the averaged simulation results. In addition, the relative standard deviation (RSD) of 10 results at each time is calculated, and then the average RSD of each time is processed, and the average RSD is used as an index to reflect the fluctuation degree of simulation results.

### 4.1. Influence of Dissemination Parameters on the Evolution Process of Public Opinion

This section starts with the parameters involved in the process of public opinion dissemination and analyzes the influence of its change on the evolution process of public opinion.

#### 4.1.1. Influence of the Proportion of Communication Individuals on the Evolution of Public Opinion at the Initial Moment

**Figure 6:**
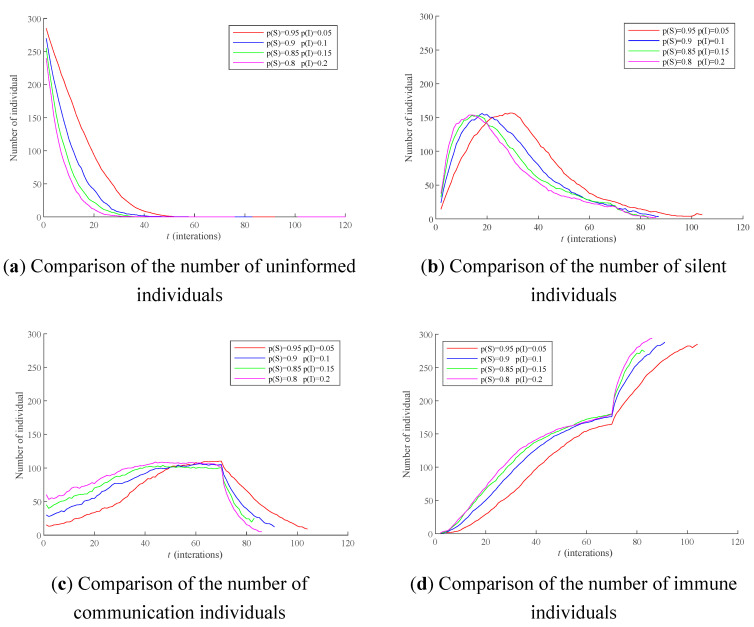
The influence of different proportions of communication individuals at the initial moment on the dissemination process of public opinion. Notes: (**a**) Comparison of the number of uninformed individuals, (**b**) Comparison of the number of silent individuals, (**c**) Comparison of the number of communication individuals, (**d**) Comparison of the number of immune individuals.

Assume that there are only two types of individuals at the initial time: Uninformed and communication individuals. Four cases of the proportion of communication individuals at the initial moment with 0.05, 0.1, 0.15, and 0.2 were selected for simulation, and the results were shown in Figure 6. The average RSD of simulation results is 5.2346%.

As can be seen from Figure 6a, the larger the proportion of communication individualsat the initial moment is, the faster the transformation speed of uninformed individuals to silent individuals and communication individuals will be in the process of public opinion dissemination. As can be seen from Figure 6b,c, no matter how the proportion of the communication individuals at the initial moment changes, the peak value of the number of silent individuals is all around 150, and the peak value of the number of communication individuals is all around 110. This shows that the proportion of the communication individuals at the initial moment only affects the dissemination speed of public opinion, but does not affect the peak value of the number of the two types of individuals. As can be seen from Figure 6d, the larger the proportion of communication individuals at the initial moment is, the faster the number of immune individuals grows in the process of public opinion dissemination. This is because the increase of the proportion of communication individuals will make the uninformed individuals receive the public opinion information earlier and turn into communication individuals or silent individuals, thus making them transform into immune individuals earlier under the effect of forgetting effect.

**Figure 7:**
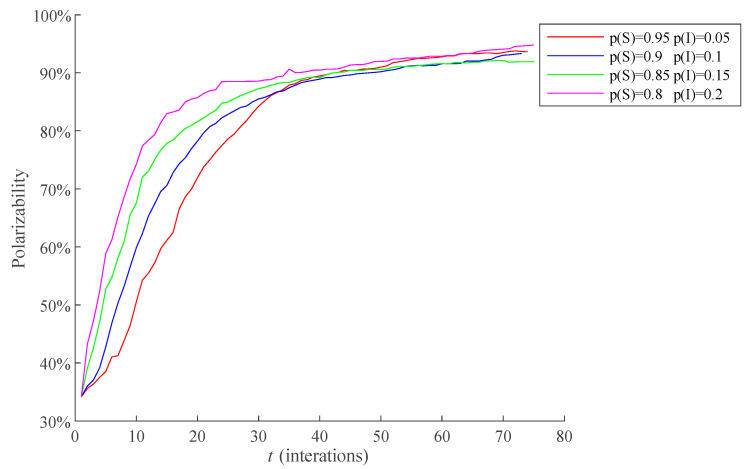
The influence of different proportion of communication individuals at the initial moment on the polarization process of public opinion.

In addition, the proportion of the communication individuals at the initial moment will not only have an impact on the process of public opinion dissemination, but also affect the process of public opinion polarization. The polarizability is defined as the proportion of individuals with extreme opinions in all individuals. The polarizability curves when the proportion of communication individuals with 0.05, 0.1, 0.15, and 0.2 at the initial moment are respectively selected for comparison, and the results are shown in Figure 7. The average RSD of simulation results is 4.0031%.

The Figure 7 shows that when 0 < *t* ≤ 20, namely the early stage of public opinion evolution, the proportion of communication individuals has a significant effect for the public opinion polarization; when *t* > 20, the influence of the proportion of communication individuals on the public opinion polarization is gradually weakened after the interaction between individuals is fully carried out. This shows that the proportion of communication individuals at the initial moment only plays an obvious facilitating role in the early stage of the public opinion evolution. This is because that most individuals have not received the public opinion information in the early stage of public opinion evolution, the number of communication individuals in this stage determines whether the public opinion can form a large-scale dissemination in a short time, and public opinion polarization is largely formed under the premise of the large-scale dissemination of public opinion. Therefore, the proportion of communication individuals at the initial moment determines the occurrence time of public opinion polarization. However, when there is sufficient interaction among individuals, almost all individuals have received public opinion information, and the influence of the proportion of communication individuals at the initial moment is no longer significant, thus making the trend of public opinion polarization gradually consistent.

According to the above analysis, in the early stage of negative public opinion events, the government should focus on controlling the number of individuals in this stage, so as to slow down the spread of public opinion information and prevent the rapid formation of public opinion polarization.

#### 4.1.2. Influence of *z*_0_ on the Evolution of Public Opinion

**Figure 8:**
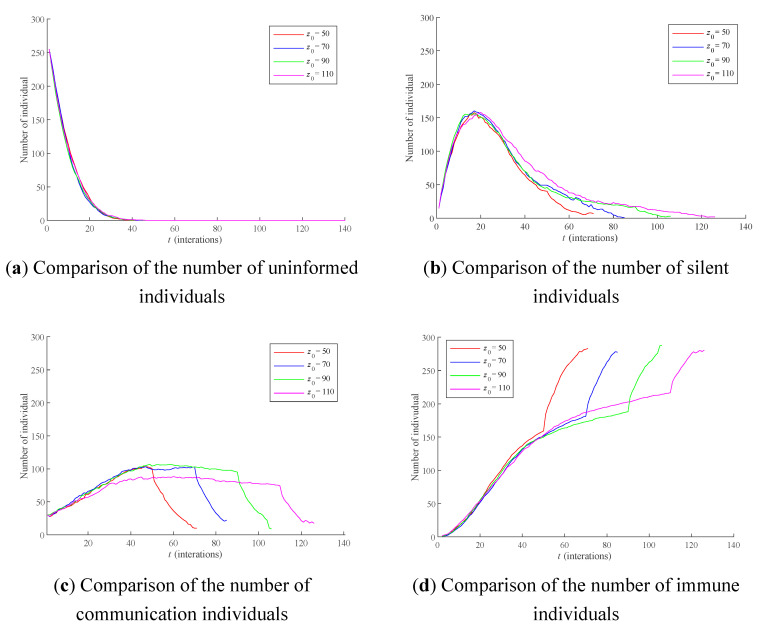
The influence of different *z*_0_ on the polarization process of public opinion. Notes: (**a**) Comparison of the number of uninformed individuals, (**b**) Comparison of the number of silent individuals, (**c**) Comparison of the number of communication individuals, (**d**) Comparison of the number of immune individuals.

To a large extent, *z*_0_ determines the influence of the forgetting effect on the process of public opinion dissemination. Here, the cases where *z*_0_ is 50, 70, 90, and 110 are respectively selected for comparison, and the results are shown in Figure 8. The average RSD of simulation results is 5.7102%.

As can be seen from Figure 8a, *z*_0_ has little influence on the number of uninformed individuals. As can be seen from Figure 8b, when *t* > 20, with the increase of *z*_0_, the transformation speed of silent individuals to communication individuals and immune individuals becomes slower. As can be seen from Figure 8c,d, when *z*_0_ is set to 50, communication individuals accelerate the transition to immune individuals at *t* > 50, and the larger *z*_0_ is, the later this time appears, leading to the longer interaction cycle between individuals.

#### 4.1.3. Influence of the *p* on the Evolution of Public Opinion

**Figure 9:**
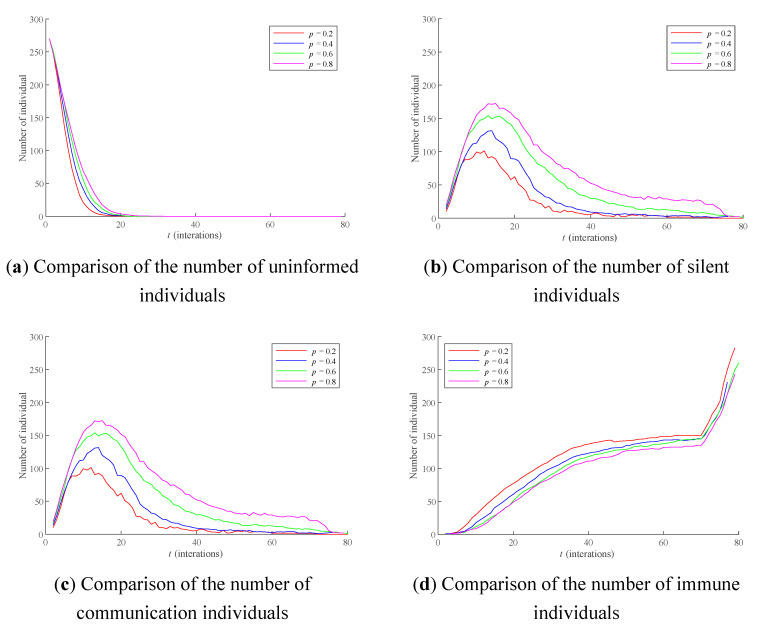
The influence of different *p* on thedissemination process of public opinion. Note: (**a**) Comparison of the number of uninformed individuals, (**b**) Comparison of the number of silent individuals, (**c**) Comparison of the number of communication individuals, (**d**) Comparison of the number of immune individuals.

**Figure 10:**
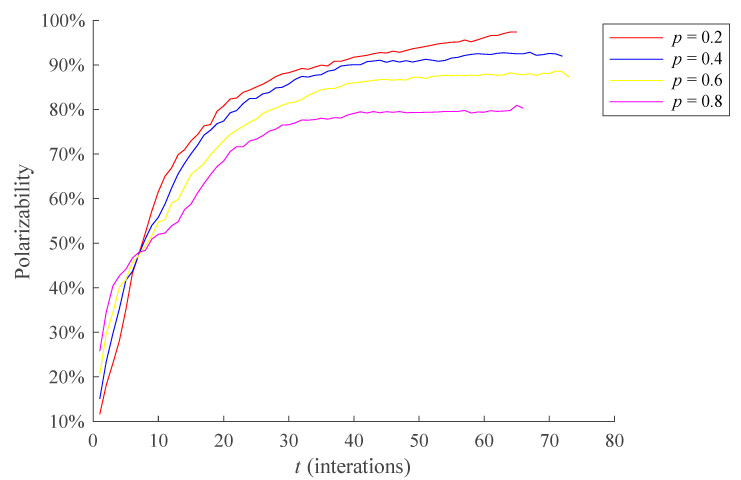
The influence of different *p* on the polarization process of public opinion.

In this section, the influence of *p* on individual state transition as well as the dissemination process of public opinion is studied. Here, individual state transitions and public opinion polarizability are compared when *p* = 0.2, 0.4, 0.6, and 0.8, respectively, and the results are shown in Figure 9 and Figure 10 below. The average RSD of simulation results are 4.4122% and 3.7061%, respectively.

As can be seen from Figure 9a–d, the larger *p* is, the slower the number of uninformed individuals in the network decreases; the more silent individuals in the network there are, and the less the numbers of communication individuals and immune individuals are. Among them, the number of silent individuals and communication individuals is particularly obvious. This is because *p* determines the difficulty of the transition between the silent individuals and the communication individuals. The greater *p* is, the more difficult it is for the silent individuals to transform into communication individuals, which makes it difficult for a large number of uninformed individuals to transform into communication individuals after receiving public opinion information.

The Figure 10 shows that when *t* ≤ 7, public opinion polarizability is larger with the increase of *p*. When *t* > 7, the smaller the *p* value is, the higher the public opinion polarizability is. This is because when *t* ≤ 7, namely at the early stage of public opinion evolution, the opinions interaction among individuals is not sufficient, and the influence of the external recognition degree of opinions on the individual communication willingness is not reflected, the expression is determined largely by the individual attitude value. The greater *p* leads to more extreme individual opinion and higher public opinion polarizability. However, when the interaction between individuals is sufficient, the external recognition degree of opinions gradually plays a decisive role in the individual communicating willingness, and the influence of individual attitude value on the communicating willingness is gradually reduced. In addition, as mentioned above, the larger *p* is, the more difficult it is for silent individuals to turn into communication individuals, and the smaller the number of communication individuals in the network is, resulting in insufficient interaction among individuals, thus leading a lower polarizability of public opinion.

### 4.2. Influence of Polarization Parameters on the Evolution Process of Public Opinion

This section starts with the parameters involved in the process of public opinion polarization and analyzes the influence of their changes on the evolution process of public opinion.

#### 4.2.1. Influence of the *T*_0_ on the Evolution of Public Opinion

**Figure 11:**
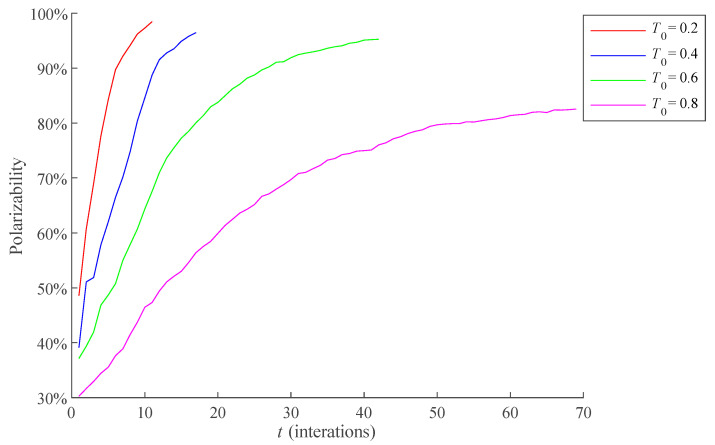
Influence of the different *T*_0_ on the polarization process of public opinion.

*T*_0_ represents the average level of individual conservative degree in the network. Different *T*_0_ will directly affect the opinion changes coefficient, which affects the network public opinion polarization trend. Therefore, under the condition that other variables remain unchanged, the influence of different *T*_0_ on the polarization process of public opinion is compared here. *T*_0_ is 0.2, 0.4, 0.6, and 0.8, respectively, and the results are shown in Figure 11. The average RSD of simulation results is 6.6981%.

As can be seen from Figure 11, when *T*_0_ is small, the formation process of public opinion polarization is shorter, and the polarizability is higher. This is because the smaller *T*_0_ is, the lower the conservative degrees of most individuals in the network are, and the more inclined most individuals are to accept others’ opinions when interacting with others, resulting in a stronger assimilation effect in the process of opinion interaction, which accelerates the formation of public opinion polarization and makes the final public opinion polarizability relatively high.

#### 4.2.2. Influence of the *γ* on the Evolution of Public Opinion Polarization

**Figure 12:**
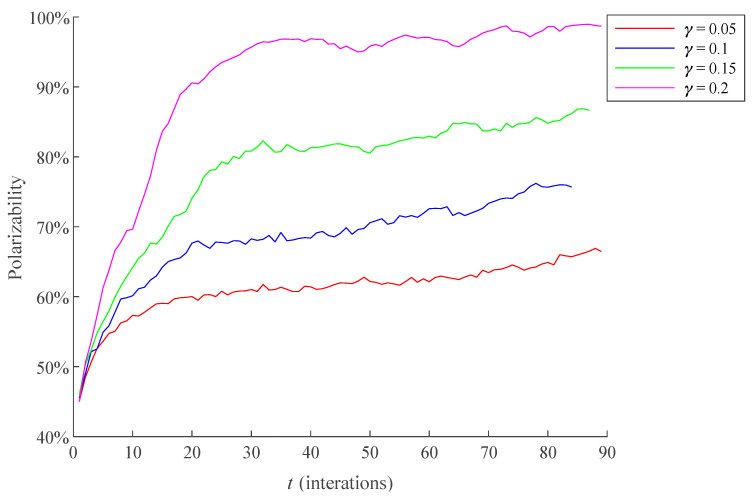
The influence of different *γ* on the polarization process of public opinion.

*γ* affects Δ*C_it_^+^*(Δ*C_it_*^−^), thus affecting the conformity of the individual to positive (negative) opinion. *γ* is 0.05, 0.1, 0.15, and 0.2, respectively, and the result is shown in Figure 12. The average RSD of simulation results is 5.9392%.

The Figure 12 shows that the smaller *γ* is, the smaller Δ*C_it_^+^*(Δ*C_it_*^−^) is, the less likely Δ*C_it_^+^*(Δ*C_it_*^−^) have an extreme value (0 or 1), which makes *C_it_^+^*(*C_it_*^−^) more moderate, namely the individual has no apparent bias forward positive opinions or negative opinions, thus inhibiting the formation of public opinion polarization.

#### 4.2.3. Combined Analysis of Polarization Parameters

**Figure 13:**
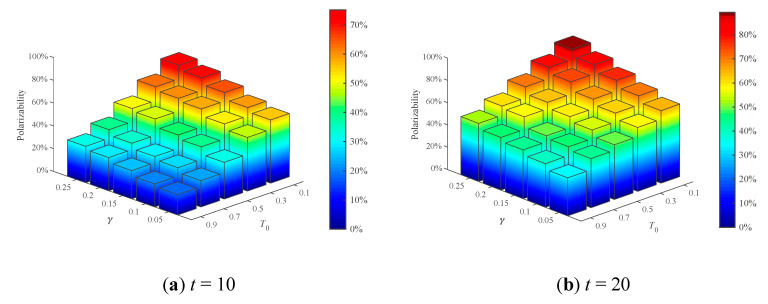

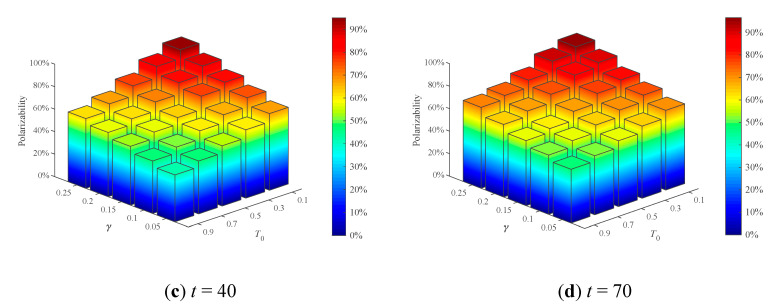
Comparison of different *T*_0_ and *γ* combination.

Through the above analysis, *T*_0_ and *γ* will affect public opinion polarization process. However, due to the urgency of the development of public opinion and the necessity of public opinion management in the real network, it is usually necessary to focus on key links to prevent further polarization of public opinion. Therefore, it is necessary to find out the key factors affecting the public opinion polarization. In this section, *T*_0_ and *γ* are combined for analysis. From the previous analysis, it can see that the polarizability of public opinion increases rapidly in the early stage of public opinion evolution, and then keeps a slow growth. Therefore, public opinion polarization when *t* = 10, 20, 40, 70 is analyzed here, and the result is shown in Figure 13. The average RSD of simulation results is 4.1341%.

Figure 13 shows that with the decrease of *T*_0_ and the increase of *γ*, public opinion polarizability increases. In addition, from Figure 13a, at *t* = 10, when *γ* is fixed, with the decrease of the *T*_0_, public opinion polarizability significantly increased; when *T*_0_ is fixed, with the increase of *γ*, public opinion polarizability rises slightly. Compared with *T*_0_, *γ* has weaker effects on the formation of public opinion polarization. It can be seen that at *t* = 10 (the early stage of public opinion evolution), the effect of individual conservative degree on the polarization of public opinion is greater than the effect of conformity, and it plays a major role in the emergence of polarization. As can be seen from Figure 13b–d, with the deepening of the interaction of opinions, the influence of individual conformity on the polarization of public opinion gradually appears. At *t* = 70 (the last stage of the evolution of public opinion), individual conformity and individual conservative degree together play a significant role in the polarization process of public opinion.

#### 4.2.4. Comparative Analysis of the Polarization Process of Public Opinion under Different Networks

**Figure 14:**
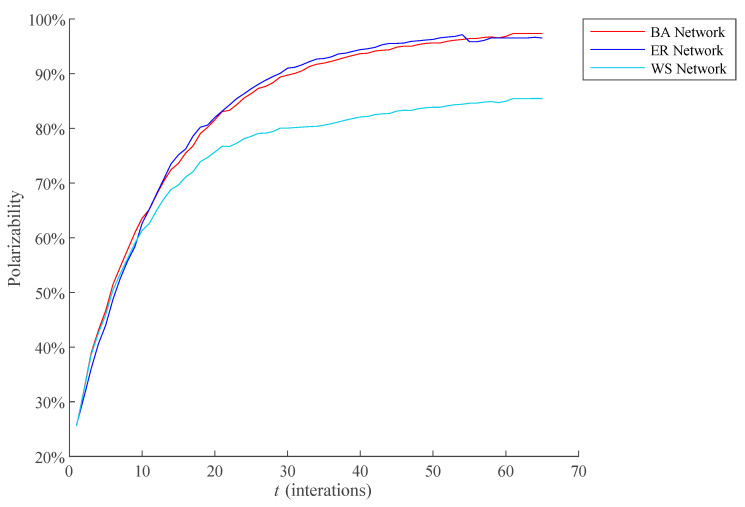
Comparison of polarizability of different networks.

Different network structures represent different ways of information exchange among individuals, which has an important impact on the public opinion polarization. Therefore, this section compared the polarization process of public opinion on the BA network, the Watts-Strogatz Network (WS network) [31], and the Erdős–Rényi Network (ER network) [32]. At the same time, in order to ensure the reliability of the simulation results, the BA network, WS network, and ER network used in the simulation need to be set as the same scale. Here, two parameters reflecting the network size are mainly considered, namely, clustering coefficient and average degree. Parameter description is shown in Table 3. The results of network comparison experiment are shown in Figure 14.

As can be seen from Figure 14, the polarization effect of public opinion under BA network and random network is similar. The polarizability is the lowest under WS network. The reasons for the above simulation results are as follows: (1) The degree distribution of BA network obeys power law distribution. A few individuals in the network have a large number of connections, and such individuals are called Hub points. A few Hub points play a leading role in the operation of the BA network. Once the public opinion information is received at the Hub in the network, the width and depth of the network public opinion dissemination will be greatly enhanced. In addition, the final effect of public opinion polarization is often determined by the dissemination degree of public opinion information in the network, while the structure of BA network determines its poor robustness in dealing with the dissemination of public opinion information. Therefore, the dissemination degree of public opinion information in the BA network is very high, and it is easy to cause obvious phenomenon of public opinion polarization. (2) In the ER network, because the connections between individuals are random, individuals can interact even if they are far away from each other. Therefore, the random network is also robust when dealing with the dissemination of public opinion information, which leads to obvious public opinion polarization. (3) The connection of most individuals in WS network is limited to the surrounding “neighbors”, which is similar to the offline interpersonal network in reality. Since such networks reduce the connection between individuals who are far away from each other, and the speed and width of information dissemination is lower than that of BA network and ER network, resulting in lower public opinion polarizability.

## 5. Empirical Analysis

In this section, the pricing of China’s self-developed COVID-19 vaccine (hereinafter referred to as vaccine pricing) is selected as a case to verify the effectiveness of the SEIR-JA model.

Since the outbreak of COVID-19, Chinese researchers have stepped up efforts to develop a vaccine against COVID-19. On 18 August 2020, Liu Jingzhen, chairman of Sinopharm Group, mentioned the pricing of China’s self-developed COVID-19 vaccine for the first time. Since then, there has been a huge public debate online about the vaccine pricing. According to the search results of Weibo topics, discussions on vaccine pricing were mainly focused on four periods: 18 August to 26 August, 23 September to 30 September, 16 October to 28 October, and 23 November to 4 December. Public opinion information related to vaccine pricing in each period is shown in Table 4.

**Figure 15:**
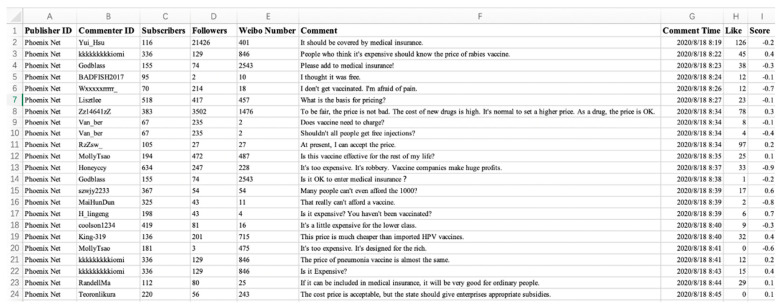
Crawl data form.

Here, 34,786 Weibo comments and relevant data during four periods are collected, and 27,194 valid data are obtained after data cleaning and scored by JIEBA [33] and emotion dictionary. The emotion of each posted comment is obtained by quantitative values, and data form is shown in Figure 15 and Table 5. Although the amount of data obtained is limited, according to the Six Degrees of Separation [34], the statistical results of these user data can reflect the universality of Weibo user behavior to a large extent.

Here, the effectiveness of the SEIR-JA model is verified from public opinion dissemination and polarization. In terms of public opinion dissemination, the SEIR model was selected and compared with the SEIR-JA model proposed in this paper to simulate the process of public opinion dissemination of vaccine pricing. In terms of public opinion polarization, J-A model was selected and compared with the SEIR-JA model proposed in this paper to simulate the process of public opinion polarization of vaccine pricing, setting the individual size in the simulation of the two models as 500. At the same time, in order to make the simulation results closer to the real situation of each time period, some parameters of three models will be adjusted according to the comment data of different time periods: (1) The proportion of the communication individuals at the initial time of the three models is equal to the proportion of the comments in the first 3 h of each time period to the total number of comments in that time period; (2) *z*_0_ in the SEIR-JA model is equal to 3/4 of the duration of each time period; (3) in the whole evolution process of public opinion events, most people have a stronger cognition of the event and the individual conformity gradually decreases. Therefore, according to the analysis results in Section 4.2.2, *ω* in the SEIR-JA model is set at 40, 45, 50, and 55, respectively, in four time periods. (4) According to the average value of comment emotion in the first 3 h of each time period, the initial attitude values of individuals in the network of four time periods were set to obey N~(−0.1,0.4), N~(−0.1,0.4), N~(0,0.4), and N~(0.2,0.4), respectively. In addition, other parameters of the SEIR-JA model were set as follows: *d*_1_ = 0.3, *d*_2_ = 0.7, *T*_0_ = 0.8, *p* = 0.5; in J-A model, other parameters are set as: *d*_1_ = 0.3, *d*_2_ = 0.7, *μ* = 0.5; in the SEIR model, the acceptance coefficient is equal to 1, the dissemination coefficient is equal to 0.3, and the immune coefficientis equal to 0.2.

**Figure 16:**
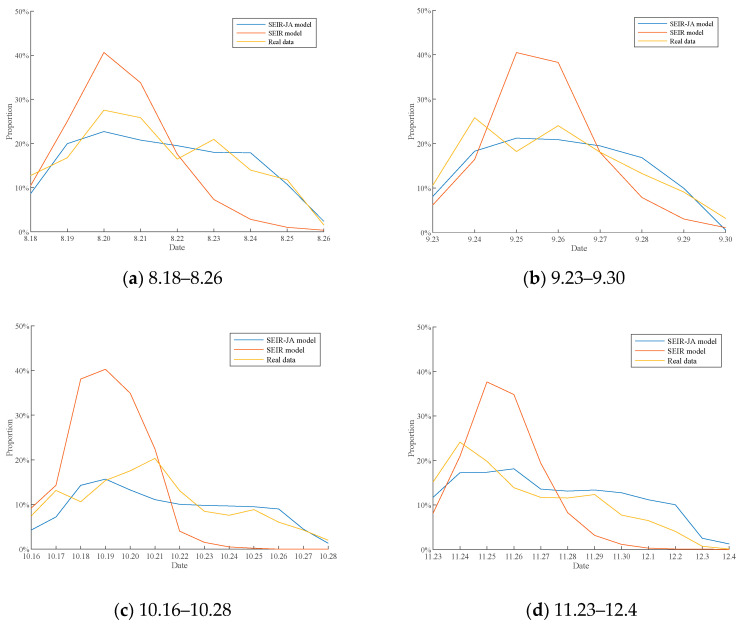
The ratio of the number of comments.

Due to the difficult acquisition of the number of uninformed and silent users, as well as the number of individuals in the network not being completely consistent with the actual number of people participating in the topic discussion, the daily number of comments cannot be directly compared. Therefore, the proportion of daily comments (the ratio of daily commented users in total commented users during the period) is set as indicators for comparison of different models of public opinion dissemination. Figure 16 compares the proportion curve of the number of comments simulated by SEIR model and SEIR-JA model with the actual curve. The average RSD of simulation results of SEIR-JA modelis 5.1134%. In the figure, the abscissa is the date, and the ordinate is the proportion of the number of comments. The blue line represents the proportion curve of the number of comments simulated by the SEIR-JA model; the red line represents the proportion curve of the number of comments simulated by the SEIR model; the yellow line shows the actual percentage of comments plotted based on comment data.

As can be seen from Figure 16a–d, the proportion curve of the number of comments simulated by the SEIR model increases rapidly at the initial stage, and then decreases rapidly after reaching the peak, and the highest proportion of the number of comments reaches about 40% in each period. In contrast, the proportion curve of the number of comments simulated by the SEIR-JA model is more flat, and the proportion of the number of comments does not show a rapid decline after reaching the peak, but remains stable for a period of time. Here, root mean square error is used to accurately reflect the error between the proportion curve of the number of comments simulated by SEIR model and SEIR-JA model and the actual proportion curve of the number of comments. The results are shown in Table 6.

In combination with Figure 16 and Table 6, it can be seen that the root mean square error of the simulation results of the SEIR-JA model in each period is smaller than that of the SEIR model, and the proportion curve of the number of comments simulated by the SEIR-JA model is closer to the actual curve, indicating that the SEIR-JA model is closer to the actual situation in terms of public opinion dissemination.

**Figure 17:**
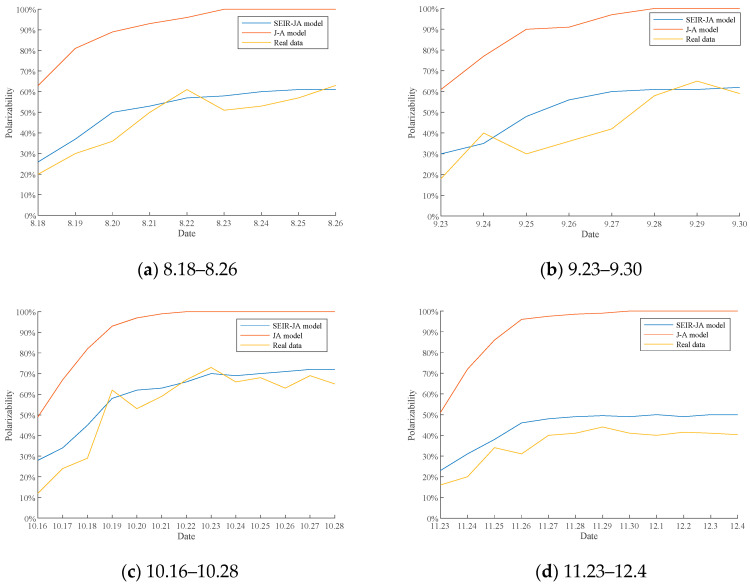
Comparison of polarizability.

In addition, in order to simulate and study the polarization process of public opinion, the scores of Weibo comments in four time periods are sorted according to the released time, and the proportion of daily extreme comments (comments with an emotional score greater than 0.8 or less than −0.8) is calculated to get the polarizability of daily comments. The polarizability curves simulated by J-A model and SEIR-JA model were compared with the actual polarizability curve, and the results were shown in Figure 17. The average RSD of simulation results of SEIR-JA model and J-A model are 4.2557% and 4.1195%, respectively. In Figure 17, the abscissa is date and the ordinate is polarizability. The blue line represents the polarizability curve simulated by the SEIR-JA model. The red line represents the polarizability curve simulated by J-A model. The yellow line represents the actual polarizability curve plotted from the comment data.

As can be seen from Figure 17a–d, the polarizability of J-A model in each periodgrows very fast and finally reaches 100%, that is, all individuals in the network hold extreme opinions. However, in the development process of public opinion events such as vaccine pricing, extreme opinions will only increase compared with the initial moment, but cannot be fully polarized. It is obviously unrealistic for the final polarizability of 100% in the J-A model. In contrast, the polarizability of the SEIR-JA model grows relatively steadily in each period, and the final polarizability stays in the range of 50–80%. Although there is a gap between it and the actual curve, the overall polarizability change trend is basically consistent with the actual situation. Therefore, the SEIR-JA model is closer to the actual situation in terms of public opinion polarization.

The above analysis shows that SEIR-JA model has a good performance in the evolution process of actual public opinion events. Therefore, the government can grasp the future evolution of public opinion events and intervene in time through SEIR-JA model.

## 6. Conclusions

In this paper, the shortcomings of SEIR model and J-A model were firstly improved, and then the improved SEIR model and J-A model were combined to build a SEIR-JA model integrating the process of public opinion dissemination and polarization. On this basis, the influence of model parameters on the evolution process of public opinion was analyzed. The following conclusions are obtained through simulation experiments:

(1) The proportion of communication individuals at the initial moment only affects the dissemination speed of public opinion, but does not affect the peak value of the number of silent individuals and communication individuals.

(2) The proportion of communication individualsat the initial moment has a significant impact on the formation of polarization in the early stage of the evolution of public opinion.

(3) At the early stage of public opinion evolution, as *p* increases, the polarizability increases accordingly. However, after sufficient interaction between individuals, the smaller *p* is, the higher the polarizability will be.

(4) At the early stage of the evolution of public opinion, the effect of individual conservative degree on the public opinion polarization is greater than the effect of conformity; with the deepening of the interaction of opinions, the influence of individual conformity on the public opinion polarization is gradually appearing. At the last stage of the evolution of public opinion, both individual conformity and individual conservatism play a significant role in the public opinion polarization.

However, the following topics need to be further explored to make up the deficiencies in this paper:

(1) For the process of public opinion dissemination, this study only focuses on the influence of individual communicating willingness and forgetting effect on public opinion dissemination. However, in reality, there are many factors that affect public opinion dissemination [35]. Therefore, subsequent studies need to consider the influence of more factors on public opinion dissemination and establish a public opinion dissemination model that is more aligned with the actual situation.

(2) This paper studies the process of public opinion dissemination and polarization only from the perspective of Internet users, while the main body of the evolution process of public opinion also includes network platforms and government [36]. Therefore, subsequent studies need to comprehensively consider the role of network platforms and government policies in the process of public opinion dissemination and polarization.

(3) Although the results of empirical analysis prove that the SEIR-JA model has a good performance for vaccine pricing and similar cases, there are still some cases that the SEIR-JA model is not suitable for. Therefore, the next work is to further optimize SEIR-JA model in the future to make it suitable for more cases.

## Figures and Tables

**Table 1:** Related parameters.

Parameter	Description	Range
*m_it_*	The external recognitiondegree of individual’s opinion at time *t*	[1, +∞)
*z*_0_	The maximum time an individual pays attention to a piece of public opinion information	[1, +∞)
*n_ij_*	Number of neighbors shared by individual *i* and *j*	(0, +∞)
*k_i_*	Number of neighbors of individual *i*	(0, +∞)
*N*	Total number of individuals in the network	(0, +∞)
*T*_0_	Average conservative degree of all individuals in the network	[0, 1]
*w_it_* ^+^	The mainstream degree of the positive opinion which individual *i* considers at time *t* (the mainstream degree of positive opinion refers to the proportion of the number of people holding positive opinions in the network)	[0, 1]
*w_it_* ^−^	The mainstream degree of the negative opinion which individual *i* considers at time *t* (the mainstream degree of negative opinion refers to the proportion of the number of people holding negative opinions in the network)	[0, 1]
*d*_1_	Assimilation threshold	[0, 1]
*d*_2_	Rejection threshold	[0, 1]
*p*	Communication threshold (refers to the critical value for individuals to express their opinions)	[0, 1]
*γ*	Change range of *w_it_*^+^ and *w_it_*^−^ per unit time	(0, 0.5]

**Table 2:** Related variables.

Variable	Description	Range
*P_i_*(*t*)	The communicating willingness of individual *i* at time *t*	[0, 1]
*z*	The time length of receiving public opinion information	[1, +∞)
*E_ij_*	Relationship strength between individuals *i* and *j*	[0, 1]
*T_i_*	Conservative degree of individual *i*	[0, 1]
*C_it_* ^+^	Conformity of individual *i* to the positive opinion at time *t*	[0, 1]
*C_it_* ^−^	Conformity of individual *i* to the negative opinion at time *t*	[0, 1]
*μ_it_*	Change coefficient of individual’s opinion at time *t*	[0, 1]
*x_i_*(*t*)	Attitude value of individual *i* at time *t*	[−1, 1]

**Table 3:** Comparison of network parameters.

Network Name	Clustering Coefficient	Average Degree
Barabási-Albert (BA) network	0.0912	48.9285
Watts-Strogatz (WS) network	0.0895	48
Erdős–Rényi (ER) network	0.0919	49.61

**Table 4:** Public opinion information in each period.

Periods	Public Opinion Information
18 August to 26 August	On 18 August, Liu Jingzhen, chairman of Sinopharm, said that the price of the COVID-19 vaccine was about 1000 Yuan for two doses.
	On 23 August, Zheng Zhongwei, director of China’s National Health Commission’s Center for Health Science and Technology Development, said vaccines can only be priced on the basis of cost, and made it clear that the final price of the vaccine would be lower than Liu’s price.
23 September to 30 September	On 23 September, Sinopharm set the basic price of COVID-19 vaccine at 600 Yuan for two doses, taking into account costs and public acceptability.
	On 25 September, Zheng said again that the guideline price of the vaccine must be within the range acceptable to the public.
16 October to 28 October	On 16 October, Jiaxing Center for Disease Control and Prevention published the instructions on COVID-19 vaccine, which mentioned that the price of the vaccine would be 400 Yuan for two doses.
	On 19 October, China’s National Medical Insurance Administration announced that preventive vaccines (including COVID-19) would not be covered by medical insurance.
	On 20 October, Shaoxing Center for Disease Control and Prevention released the guidelines for emergency vaccination of COVID-19 vaccine in fall and winter, which mentioned that the price of the vaccine is 200 Yuan per dose.
23 November to 4 December	On 23 November, Liang Zongan, a professor at West China Hospital of Sichuan University, said in an interview that the price of COVID-19 vaccine in Sichuan is the same as that in Zhejiang, at 200 Yuan per dose.

**Table 5:** Comments information.

Periods	Comments	Users	Average Comment Emotion in First Three Hours	Duration
18 August to 26 August	3665	1961	−0.11	9 days
23 September to 30 September	6620	3042	−0.13	8 days
16 October to 28 October	11,871	6785	0.05	13 days
23 November to 4 December	5038	2735	0.21	12 days

**Table 6:** Root mean square error.

	18 August to 26 August	23 September to 30 September	16 October to 28 October	23 November to 4 December
SEIR-JA model	2.8802	4.4582	3.7955	3.9768
SEIR model	9.6939	10.4669	12.4072	9.4221

## Data Availability

The data used to support the findings of this study are available from the corresponding author upon request.
